# A systematic review of the quality of reporting of interventions in the surgical treatment of Crohn’s anal fistula: an assessment using the TIDiER and Blencowe frameworks

**DOI:** 10.1007/s10151-020-02359-7

**Published:** 2021-02-18

**Authors:** S. Tyrell, E. Coates, Steven R. Brown, M. J. Lee

**Affiliations:** 1 School of Health and Related Research, University of Sheffield, Sheffield, UK; 2 Academic Directorate of General Surgery, Sheffield Teaching Hospitals NHS Foundation Trust, Sheffield, UK; 3 Department of Oncology and Metabolism, The Medical School, University of Sheffield, Sheffield, S10 2RX UK

**Keywords:** Crohn’s disease, Anal fistula, Reporting methodology

## Abstract

**Background:**

Crohn’s anal fistula is a challenging condition, and may require multiple surgical procedures. To replicate successful procedures, these must be adequately reported in the literature. The aim of this study was to review the quality of reporting of components of surgical interventions for Crohn’s anal fistula.

**Methods:**

A systematic review was conducted. It was registered with PROSPERO (CRD:42019135157). The Medline and EMBASE databases were searched for studies reporting interventions intended to close fistula in patients with Crohn’s disease, published between 1999 and August 2019. Abstracts and full texts were screened for inclusion by two reviewers. Dual extraction of data was performed to compare reporting to the TIDiER and Blencowe frameworks for reporting of interventions.

**Results:**

Initial searches identified 207 unique studies; 38 full texts were screened for inclusion and 33 were included. The most common study design was retrospective cohort (17/33), and the most frequently reported interventions were anal fistula plug (*n *= 8) and fibrin glue (*n *= 6). No studies showed coverage of all domains of TIDieR. Reporting was poor among domains related to who provided an intervention, where it was provided, and how it was tailored. Reporting of domains in the Blencowe framework was poor; the majority of studies did not report the component steps of procedures or efforts to standardise them.

**Conclusions:**

This study demonstrates that reporting on technical aspects of interventions for Crohn’s anal fistula is poor. Surgeons should aim to improve reporting to allow accurate reproduction of techniques both in clinical practice and in clinical trials.

**Supplementary Information:**

The online version of this article (10.1007/s10151-020-02359-7) contains supplementary material, which is available to authorized users.

## Introduction

Crohn’s disease is a chronic inflammatory condition, and can have effects anywhere within the gastrointestinal tract. It is typically treated using a range of drugs that have effects on the immune system [1]. However, some presentations of Crohn’s disease require significant elements of management by a surgeon. Prevalent among these is Crohn’s anal fistula. This is a condition with considerable morbidity, and will require repeated surgical interventions as long-term remission is achieved in as few as 30% of patients [2].

There are a range of potential surgical techniques currently in use [3]. However, there is no clear front running intervention to achieve the ‘Holy Grail’ of fistula closure [4]. There are three main components within a study which assesses a surgical intervention; the population who were studied, the intervention or treatment(s) compared, and the outcomes reported. A mismatch between studies in any of these components has the potential to introduce heterogeneity into an analysis, potentially leading researchers to an incorrect conclusion. A particular perceived challenge to replication of research findings in practice is the fidelity with which one is able to reproduce interventions from a study [5].

One method to address limited reporting on the conduct of interventions is to use a reporting checklist. There are two such checklists available at present. The first of these is the Template for Intervention Description and Replication (TIDieR) checklist [6]. This is a 12-item checklist, which seeks details of interventions including mode and timing of delivery. TIDieR is a more generic framework, and is easily adaptable to medical and more complex interventions beyond surgery [7, 8]. The Blencowe framework for surgical interventions is broadly broken into three domains: intervention description, standardisation of description, and monitoring the fidelity of the intervention [5]. As a tool designed specifically for surgical interventions, this identifies domains that might not be addressed through TIDieR. A summary of these tools is presented in Table 1.

**Table 1:** Summary of TIDieR and Blencowe frameworks

Item number	TIDieR item	Blencowe section	Blencowe Iiem
1	Brief name of the surgical intervention	Section 1: Intervention description	
2	Why is the surgical intervention being carried out?	1.1	Overall technical purpose of the intervention
3	What materials have been used in the intervention?	1.2	Identification of the intervention components
4	What procedures have been carried out?	1.3	Identification of individual steps of the intervention
5	5 Who provided each category of the intervention?	Section 2: Standardisation of surgical interventions	
6	6 How was the intervention carried out?	2.1	Types of standardisation
7	7 Where was the intervention completed?	2.2	Conditions relating to standardisation
8	When and how much? The number of times the intervention was delivered and over what period of time.	2.3	Flexibility of standardisation
9	Tailoring; was the intervention planned to be personalised?		
10	Was the intervention modified through the duration of the study?		
11	How well was the intervention planned?		
12	If adherence to the plan was assessed, how well did the intervention go according to this plan?	Section 3: Fidelity	
		3.1	Deviation from intervention
		3.2	Deviation from the components
		3.3	Deviation from steps

The aim of this study was to provide evidence on the quality of surgical reporting for the treatment of fistula formation in patients with Crohn’s disease using the TIDieR and Blencowe frameworks.

## Materials and methods

### Overview and registration

This systematic review was conducted with reference to the Cochrane Handbook [9], and is reported in line with the Preferred Reporting Items for Systematic Reviews and Meta-Analyses: (PRISMA) guidelines [9, 10]. It was prospectively registered on the PROSPERO database (CRD:42019135157).

### Inclusion and exclusion criteria

Studies reporting the use of surgical procedures to achieve fistula closure in Crohn’s anal fistula were eligible for inclusion. Surgical procedures were defined as those performed by surgeons in an operating theatre. Eligible study designs included case series, cohort studies, and randomised-controlled trials. Studies had to be published after 1999, as this approximates the beginning of use of anti-TNF drugs in this setting [11]. Studies published until August 2019 were eligible for inclusion. This decision was made as anti-TNF drugs have moderate effects on remission and maintenance of fistulating disease [1]. They can conceivably change the surgical decision-making in this setting based on likelihood of recurrence. No restrictions were applied based on reported outcomes, and studies had to be published in English.

Studies reporting on procedures for cryptoglandular or traumatic fistulae were not eligible for inclusion, unless they reported approaches to Crohn’s and other aetiologies separately. As a seton might be considered to be a drainage or temporising procedure rather than one aimed at closure, it was not eligible for inclusion in this study.

### Search strategy and information sources

Scoping searches of Google Scholar and the National Center for Biotechnology Information were undertaken to identify relevant Medical Subject Heading (MeSH) terms. These were used to develop a search strategy which was deployed into EMBASE and MEDLINE databases via the OVID interface. Search strategies are presented in Online Appendix A. A secondary hand search of references of included papers was also performed.

### Selection of studies

Candidate abstracts were assessed for inclusion against the eligibility criteria by two reviewers (ST and ML). Full manuscripts of eligible abstracts were retrieved and screened again by a single reviewer, with queries resolved by a second reviewer.

### Data extraction

Data were extracted from the final selection of studies into a pre-designed proforma using Microsoft Excel (Microsoft, Redmond, VA, USA). Data extraction retrieved study descriptors, participant descriptors, and nature of outcome used. Intervention name was taken directly from the included study. Where a global name or label was not provided in a study, the intervention was reviewed by the research team to classify it.

The following items were assessed in the TIDieR framework, with reference to the source document [6] for definitions of each item: name of surgical intervention, why it was carried out, what materials were used for the intervention, what procedures were carried out, who provided each category of the intervention, how was the intervention carried out, when and how many times the intervention was performed, tailoring of the intervention, how the intervention was modified during the study, how well was the intervention planned, and how was adherence to the plan assessed?.

The following domains of the Blencowe framework were investigated, with reference to the source document [5] for guidance on their use: overall technical purpose of the intervention, identification of the intervention components, identification of individual steps of the intervention, types of standardisation, conditions relating to standardisation, flexibility of standardisation, and monitoring of the fidelity of the intervention. Data extraction was performed independently by two reviewers (ST and EC).

### Risk of bias

Risk of bias was assessed using either the Cochrane Risk of Bias (ROB) tool [12] or the Risk of Bias In Non-randomised Studies of Interventions (ROBINS-I) tool [13]. Assessment was performed by two independent reviewers (ST and EC).

### Synthesis of results

A descriptive synthesis of concordance with the two reporting frameworks was undertaken. A descriptive comparison of coverage of domains within each framework was made. Correlation between reporting coverage (total number of domains covered) in Blencowe and TIDieR frameworks was carried out using Spearman’s correlation. Significance was set at 0.05 a priori. Analyses were conducted using R Studio software (RStudio team, Boston, MA) using Tidyverse [14].

## Results

### Search results

**Fig. 1:**
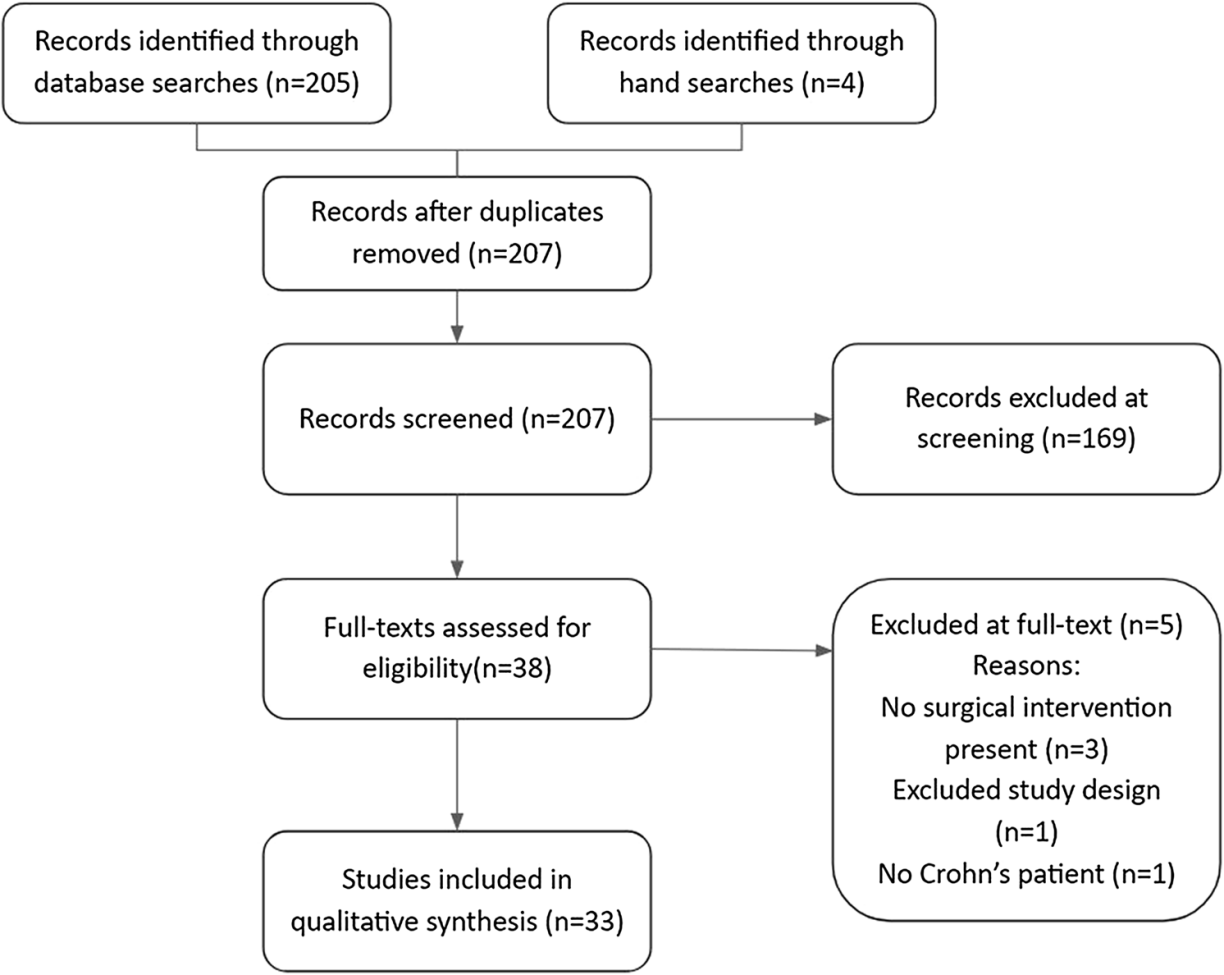
PRISMA flowchart

Searches of databases identified 205 results, and four further studies were identified through hand searches of references. After removal of duplicates, this left 207 studies to be screened. Of these, 169 were excluded and full texts were retrieved for 38 studies. Of these 38, 5 were excluded at full-text review (no surgical intervention performed *n *= 3, inappropriate study design *n *= 1, and non-Crohn’s fistula *n *= 1). This left 33 studies for inclusion in the review. This is summarised in the PRISMA flowchart (Fig. 1).

### Characteristics of studies

Of the 33 included studies, 3 were randomised-controlled trials (RCTs) [15–17], 9 were prospective cohort studies [18–26], 17 were retrospective cohort studies [27–44],2 were phase 1 clinical trials [45, 46], and 1 study was a phase 2 clinical trial [47]. Fourteen of the studies included only Crohn’s patients in their investigation, with the remainder treating a mix of Crohn’s and cryptoglandular fistula. Eleven studies originated in the United States of America [20, 24–27, 32, 36, 40, 42, 43, 46], and 5 each in France [15, 17, 35, 41, 44] and Germany [19, 21–23, 29, 31]. The median number of Crohn’s fistula patients in a study was 14, range from 3 [33] to 212 [16] (Table 1). No studies reported use of either of the reporting frameworks under review here.

### Interventions studied

Ten interventions were identified. These were anal fistula plug (AFP) (*n *= 8) [15, 22–24, 33, 34, 36, 46], fibrin glue (*n *= 6) [17, 25, 35, 40–42], advancement flap (*n *= 4) [26, 32, 39, 43], fistulotomy (*n *= 3) [28, 37, 38], over-the-scope clip (OTSC) (*n *= 3) [29, 31, 44], video-assisted anal fistula treatment (VAAFT) (*n *= 2) [18, 21], adipose-derived stem cells (ASCs) (*n *= 3) [16, 45, 47], ligation of intersphincteric fistula track (LIFT) (*n *= 2) [20, 27], fistula tract laser closure (FiLAC) (*n *= 1) [19], and cone-like resection (CLR) (*n *= 1) [30]. Three studies included more than one intervention [34, 37, 39] (Table 2).

**Table 2:** Characteristics of included studies

Author	Country	Study type	No. of Crohn’s patients	Intervention
Senejoux et al. (2015)	France	RCT	106	AFP
Cheung et al. (2018)	UK	Prospective cohort	7	VAAFT
Kaminski et al. (2017)	USA	Retrospective cohort	23	LIFT
Wilhelm et al. (2017)	Germany	Prospective cohort	13	FiLAC
Dietz et al. (2017)	USA	Phase 1 clinical trial	12	AFP
Papaconstantinou et al. (2017)	Greece	Retrospective cohort	59	Fistulotomy
Prosst et al. (2016)	Germany	Retrospective cohort	8	OTSC
Mattioli et al. (2015)	Italy	Retrospective cohort	11	Cone-like resection
Mennigen et al. (2015)	Germany	Retrospective cohort	6	OTSC
Gingold et al. (2014)	USA	Prospective cohort	15	LIFT
Lee et al. (2013)	Korea	Phase 2 study	50	ASC
Schwander (2013)	Germany	Prospective cohort	13	VAAFT
Jarrar et al. (2011)	USA	Retrospective cohort	33	Advancement Flap
Owen et al. (2010)	Australia	Retrospective cohort	3	AFP
Chung et al. (2010)	Canada	Retrospective cohort	40	AFP
Grimaud et al. (2010)	France	RCT	77	Fibrin Glue
de Parades et al. (2010)	France	Retrospective cohort	11	Fibrin Glue
de la Portilla et al. (2013)	Spain	Phase I/IIa clinical trial	24	ASC
Safar et al. (2009)	USA	Retrospective cohort	4	AFP
van Koperen et al. (2009)	Netherlands	Retrospective cohort	61	Fistulotomy
Schwander et al. (2009)	Germany	Prospective cohort	16	AFP
Davies et al. (2008)	UK	Retrospective cohort	18	Fistulotomy
Schwander et al. (2008)	Germany	Prospective cohort	7	AFP
O’Connor et al. (2006)	USA	Prospective cohort	20	AFP
van der Hagen et al. (2006)	Netherlands	Retrospective cohort	21	Advancement Flap
Vitton et al. (2005)	France	Retrospective cohort	14	Fibrin Glue
Sentovich (2003)	USA	Retrospective cohort	5	Fibrin Glue
Sentovich (2001)	USA	Retrospective cohort	4	Fibrin Glue
Nelson et al. (2000)	USA	Retrospective cohort	17	Advancement Flap
Park et al. (2000)	USA	Prospective cohort	2	Fibrin Glue
Hyman (1999)	USA	Prospective cohort	14	Advancement Flap
Gautier et al. (2015)	France	Retrospective cohort	6	OTSC
Panes (2016)	Spain	RCT	212	ASC

*AFP* Anal fistula plug, *VAAFT* video-assisted anal fistula treatment, *FiLAC* fistula laser closure, *OTSC* Over the scope clip, *LIFT* Ligation of intersphincteric tract, *ASC* adipose stem cell, *RCT* Randomised controlled trial

### Risk of bias

Non-randomised studies: The assessment of bias using this tool is presented in Supplementary Table S1a. The 30 non-randomised studies demonstrated the issues including risks of bias due to confounding, selection of patients, outcome measurements, and reporting of results. Deviations from intervention were also not assessed. Bias in the 3 randomised trials was generally considered to be low, although there were some concerns related to blinding of those administering the intervention. Summaries of bias assessment are presented in Online Appendix B.

### Synthesis of results

#### TIDieR framework

Assessment using the TIDieR checklist found that all studies reported intervention name (item 1), rationale (item 2), how the surgery was carried out (item 6), and when and how much (item 8). Item 3, the materials that were used in the intervention, showed higher levels of reporting with 31/33 (93.9%) of papers including a list of materials used. Though a large proportion of studies listed the items that were used, this was to a varying standard. An example is seen is van Koeperen et al. [37] and Davies et al. [38], which reported only general or locoregional anesthetic, and barium as items. Most other studies reported an extensive list of items used. The majority of studies also reported the procedure that was undertaken on the participants [31/33 (93.9%)]. The standard to which these were reported varied however. Some papers included the description of their procedure in other locations such as previous papers as in Kaminski et al. 2017 [27]. Other studies provided significantly greater detail within the paper such as in Mennigen et al. 2015 [31] and Wilhelm et al. 2017 [19].

Only 20/33 (60.6%) of the included manuscripts provided any description of who carried out the different stages of the intervention (item 5). Data were extracted for this item if an occupation and role in the study were provided. Examples were clear in the studies where this was reported. This is highlighted well in a paper on FiLAC which stated ‘One experienced colorectal surgeon carried out the procedure. A sonographer experienced in endoluminal and ultrasound conducted the preoperative clinical examination**’ [**19]. Item 7, where these interventions are carried out, was reported in 23/33 (69.7%). Tailoring of intervention (item 9) was reported in 21/33 (63.6%) of studies. Tailoring was present in the study if any personalisation of the procedure occurred; twelve studies showed no evidence of reporting this item at all. Examples extracted include that seen in Papaconstantinou et al. [28] where the intervention used depended on the fistula anatomy among other characteristics such as presence of proctitis, providing a tailored approach. Item 10 is modifications of the intervention, which typically occurs with learning. Descriptions for this item were reported in 5/33 (15.1%). Whilst this was poorly reported, some studies reported this well. For example, Hyman et al. evidenced a modification in detail [26]. In this study, bowel confinement with opiates was omitted towards the end, having been present in the early stages. Item 11, strategies to improve fidelity/adherence, was reported in 6/33 (18.2%) studies. This was very poorly reported as the few studies that evidenced the inclusion of a plan were not detailed. An example of this is in Owen et al. [33], where it is stated that the ‘Cook Surgisis AFP was inserted according to the Cook guidelines’, but does not elaborate further. No studies reported the actual fidelity of intervention delivery (item 12). A summary of reporting within this framework is presented in Table 3.

**Table 3:** Reporting according to TIDieR framework

	TIDieR item number											
	1	2	3	4	5	6	7	8	9	10	11	12
Anal fistula plug												
Senejoux et al. (2015)	X	X	X	X	X	X	X	X	X	–	–	–
Dietz et al. (2017)	X	X	X	X	X	X	X	X	–	–	–	–
Owen et al. (2010)	X	X	X	X	–	X	–	X	–	–	X	–
Chung et al. (2010)	X	X	X	X	X	X	X	X	–	–	–	–
Safar et al. (2009)	X	X	X	X	–	X	–	X	–	X	X	–
Schwander (2013)	X	X	X	X	X	X	X	X	X	–	–	–
Schwander et al. (2008)	X	X	X	X	–	X	X	X	X	–	–	–
O’Connor et al. (2006)	X	X	X	X	–	X	–	X	X	–	–	–
Video-assisted anal fistula treatment												
Cheung et al. (2018)	X	X	X	X	–	X	X	X	–	–	–	–
Schwander et al. (2009)	X	X	X	X	–	X	X	X	X	–	–	–
Ligation of intersphincteric fistula tract												
Kaminski et al. (2017)	X	X	–	–	X	X	–	X	–	–	–	–
Gingold et al. (2014)	X	X	X	X	X	X	X	X	X	–	–	–
Fistula laser closing (FiLaC)												
Wilhelm et al. (2017)	X	X	X	X	X	X	X	X	X	–	–	–
Fistulotomy												
Papaconstantinou et al. (2017)	X	X	X	X	X	X	–	X	X	–	–	–
van Koperen et al. (2009)	X	X	X	X	–	X	–	X	X	–	–	–
Davies et al. (2008)	X	X	X	X	X	X	–	X	X	–	–	–
Over- the- Scope Clip												
Prosst et al. (2016)	X	X	X	X	X	X	X	X	X	–	–	–
Mennigen et al. (2015)	X	X	X	X	–	X	X	X	X	–	–	–
Gautier et al. (2015)	X	X	X	X	X	X	–	X	–	–	X	–
Cone-like resection												
Mattioli et al. (2015)	X	X	X	X	–	X	–	X	–	–	–	–
Advancement flap												
Jarrar et al. (2011)	X	X	X	X	X	X	X	X	X	–	X	––
van der Hagen et al. (2005)	X	X	–	–	–	X	X	X	X	–	–	–
Nelson et al. (2000)	X	X	X	X	X	X	–	X	X	X	–	–
Hyman (1999)	X	X	X	X	X	X	X	X	–	X	X	–
Fibrin glue												
Grimaud et al. (2010)	X	X	X	X	X	X	X	X	X	–	–	–
de parades et al. (2010)	X	X	X	X	–	X	X	X	X	–	–	–
Vitton et al. (2005)	X	X	X	X	X	X	X	X	–	–	X	–
Sentovich (2003)	X	X	X	X	X	X	X	X	–	X	–	–
Sentovich (2001)	X	X	X	X	X	X	X	X	X	–	–	–
Park et al. (2000)	X	X	X	X	–	X	X	X	X	–	–	–
Adipose-derived stem cells												
Lee et al. (2013)	X	X	X	X	–	X	X	X	X	–	–	–
de la Portilla et al. (2013)	X	X	X	X	X	X	X	X	X	–	–	–
Panés et al. (2016)	X	X	X	X	X	X	X	X	–	X	–	–

#### Blencowe framework

Item 1.1 of the Blencowe framework, technical purpose of the intervention, was reported in all but one study (32/33, 96.9%). The one study that did not report this item was Davies et al. Whilst the surgical procedure was named, no indication of the technical purpose of the procedures (symptomatic relief/fistula closure at internal or external opening) was given. Item 1.2, identification of intervention components, was reported in 29/33 (87.8%). Item 1.3, identification of individual steps involved in the procedure, was reported by only 12/33 (36.3%) studies. This was notably absent for all studies reporting use of the LIFT procedure, fistulotomy, and fibrin glue-based interventions. Commonly, papers would report the basic components of the intervention as listed in the Blencowe framework,; however, some individual steps were missing (17/33, 51.5%). For example, the study on fistula plug use by O’Connor et al. provides detail on the placement of a fistula plug, but provides superficial detail on the tract preparation required to facilitate this [24]. Item 2.1, types of standardisation, were reported in 14/33 (42.4%) studies. The conditions relating to standardisation, i.e., operative or patient conditions that altered the procedure (item 2.2), were reported in 11/33 (33.3%). Flexibility of standardisation, i.e., allowing use of different sutures or tools based on preference, was reported in 9/33 (27.2%) studies. Though some studies included evidence of standardisation (items 4-6), this was not consistent. For example, in their study on VAAFT, Schwandner et al. reported standardisation of antibiotic prophylaxis, and flap formation [21]. Details on the performance of the video-assisted component (cautery settings, duration, etc.) were not presented. This pattern of incomplete reporting of standardisation was seen across studies.

The most poorly reported components were the items that were included in Sect. 3 of the Blencowe framework, fidelity of the intervention. The levels of fidelity were poorly reported; deviation from the intervention (3.1) 2/33 (6.1%), deviation from the components reported (3.2) in 6/33 (18.2%), and deviation from the steps (3.3) in 0/33. Any form of deviation from the individual steps of the intervention was not reported in any of the studies. Nelson et al. were the only authors to report on both 3.1 and 3.2, noting that they abandoned the use of the combined surgical method and deviated from the intended intervention [43]. They also deviated from intended components by adopting mechanical bowel cleansing increasingly towards the later stages of the study. A summary of reporting is presented in Table 4.

**Table 4:** Reporting according to Blencowe framework

Paper	Blencowe item number								
	1	2	3	4	5	6	7A	7B	7C
Anal fistula plug									
Senejoux et al. (2015)	X	X	X	–	–	–	–	–	–
Dietz et al. (2017)	X	X	X	X	–	–	X	–	–
Owen et al. (2010)	X	X	–	–	–	–	–	–	–
Chung et al. (2010)	X	X	–	–	–	–	–	–	–
Safar et al. (2009)	X	X	–	X	–	X	–	X	–
Schwander (2013)	X	X	X	X	X	X	–	–	–
Schwander et al. (2008)	X	X	X	X	X	X	–	–	–
O’Connor et al. (2006)	X	X	–	X	X	X	–	–	–
Video-assisted anal fistula treatment									
Cheung et al. 2018	X	X	X	–	–	–	–	–	–
Schwander et al. 2009	X	X	X	X	X	X	–	–	–
Ligation of intersphincteric fistula tract									
Kaminski et al. (2017)	X	–	–	–	–	–	–	–	–
Gingold et al. (2014)	X	X	–	–	–	–	–	–	–
Fistula Laser Closing									
Wilhelm et al. (2017)	X	X	X	X	X	–	–	–	–
Fistulotomy									
Papaconstantinou et al. (2017)	X	X	–	X	X	–	–	–	–
van Koperen et al. (2009)	X	–	–	X	X	–	–	–	–
Davies et al. (2008)	–	–	–	–	–	–	–	–	–
Over-the-scope clip									
Prosst et al. (2016)	X	X	X	X	–	–	–	X	–
Menningen et al. (2015)	X	X	X	X	X	X	–	–	–
Gautier et al. (2015)	X	X	–	–	–	–	–	–	–
Cone-like resection									
Mattioli et al. (2015)	X	X	–	–	–	–	–	–	–
Advancement flap									
Jarrar et al. (2011)	X	X	X	X	X	–	–	–	–
van der Hagen et al. (2005)	X	–	–	X	X	X	–	–	–
Nelson et al. (2000)	X	X	–	–	–	–	X	X	–
Hyman (1999)	X	X	–	–	–	–	–	X	–
Fibrin glue									
Grimaud et al. (2010)	X	X	–	–	–	–	–	–	–
de parades et al. (2010)	X	X	–	–	–	–	–	–	–
Vitton et al. (2005)	X	X	–	–	–	–	–	–	–
Sentovich (2003)	X	X	–	–	–	–	–	X	–
Sentovich (2001)	X	X	–	–	–	X	–	–	–
Park et al. (2000)	X	X	–	–	–	–	–	–	–
Adipose-derived stem cells									
Lee et al. (2013)	X	X	X	X	X	X	–	–	–
de la Portilla et al. (2013)	X	X	X	–	–	–	–	–	–
Panés et al. (2016)	X	X	–	–	–	–	–	X	–

## Discussion

This study has assessed the reporting of the technical conduct of several surgical interventions aimed at definitive fistula treatment in Crohn’s disease. It demonstrates a wide variation in the quality of reporting of interventions, with no intervention achieving 100% coverage in any study.

One of the more notable areas where information was missing was across the domain of tailoring and standardisation. There is potential for tailoring to occur in most scenarios; this could be the selection of suture size or material, the need to modify an incision due to sphincter proximity, or how to address any residual sepsis. There is also the potentially concerning lack of detail on the exact nature of the procedure received, i.e., fidelity. It is not clear if all participants received the intervention, they were expected to have, or whether other procedures were involved. It is easy to see how this could introduce bias in results. The TIDieR framework also points out the need to define who is delivering a procedure. In a surgical context, this is important in terms of learning curves, i.e., experience leads to improved outcomes [48]. Data from UK practice suggest that interventions in Crohn’s fistula are typically delivered by consultant surgeons [3], and whilst this might be inferred in these reported studies, it is not explicit.

This review shows that coverage of higher level domains (name of procedure and broad aim of procedure) is frequently performed. More specific details such as fidelity and tailoring are poorly reported. It has also shown that there is variation in the quality of reporting, where retrospective studies report fewer operative details than prospective studies. This is not surprising, as the retrospective study dataset may be lacking contemporaneous detail. The finding of no difference in numbers reported according to other study designs might reflect low numbers of RCTs in this review, leading to false-negative results. We have attempted to identify comparable studies to the one presented here, but searches of citations of the original TIDieR and Blencowe documents revealed no similar surgically based work.

It is widely accepted that the rates of success of surgical interventions in Crohn’s anal fistula can be disappointing. It is also notable that the rates of healing reported in surgical studies can vary widely. For example, reported ranges are from 15 to 86% for fistula plug and 29 to 79% for stem cell treatment [4]. The stem cell trial is worthy of specific comment [16]. Interventions were explained in detail with a robust attempt at standardisation. The addition of a standardised control arm is widely attributed to the relatively high efficacy of this arm compared with other trials. Broadly speaking, three main factors might be driving this variation. Either, there are patients with different disease characteristics going into studies [49], or there is a lack of consistency in the reporting of interventions, or there is variation in the measurement of outcomes [50]. The variation in quality of intervention reporting between a study such as Panes et al. [16] vs others with poorer reporting provides some insight into how limited reporting of interventions might be influencing the relative effectiveness of interventions in this study.

The challenges related to reporting quality of interventions is not limited to this setting, and have been seen across surgical and related specialties [8, 51, 52]. It is recognised that much of the surgical literature is made up of cohort studies. Where these are retrospective in nature, and particularly where they are large studies, it is likely to be difficult to extract all this information from historic records. If it is possible to retrieve relevant data, journal reporting restrictions (e.g., article length) might make it difficult to report all variations. It is, however, important to ensure coverage of key domains, so that ‘active’ ingredients of procedures can be appropriately replicated in wider practice. Some clinicians and researchers are averse to the idea of standardisation of procedures. There is debate about the level at which standardisation should happen [53], particularly as we move towards personalised or precision approaches to conditions [54]. Reporting of any and all deviations from treatment plans, no matter how minor, might lead to overload of potentially irrelevant information. Despite these challenges, accurate reporting is essential to allow the community to accurately determine the effectiveness (or not) of surgical treatments for this debilitating condition.

The study does have several methodological strengths. The authors are reassured about the robustness of the screening process, and have compared the identified studies to other reviews and have not identified any key missing references. It was prospectively registered, used a robust search strategy, dual extraction, and bias assessment, and used two tools to assess quality of reporting. This study is not without limitations. Regarding the assessment tools themselves, TIDieR is designed to be used across multiple intervention types (medical, surgical, physiotherapy, etc.) [6]. As a result, it has broad domains with quite broad descriptors. This can lead to challenges in its use in settings such as specific surgical procedure and subsequent loss of clarity in reporting some aspects. The Blencowe framework was developed with the intention to use it in randomised trials [5]. The majority of surgical studies used here were cohorts and often retrospective in nature. Extracting the relevant items for all of the domains in this framework is challenging, and may be borderline impossible in some situations. Therefore, it may be more appropriate to limit the use of this tool to prospective cohort studies. The authors did consider the use of the CONSORT-NPT extension as an additional assessment framework [55]. This was ultimately dismissed as it was felt that the Blencowe and TIDieR tools were more relevant to the multiple non-randomised trial-based designs.

This study highlights some important findings for researchers. If colorectal surgeons wish to offer the best surgical interventions for their patients, the quality of reporting in studies should also improve. Efforts have already been made to standardise outcome reporting for this condition [56]. Whilst it is not fair to judge historic papers against standards that were developed after their completion, it is fair to apply these standards to the future. Surgeons should focus on capturing and reporting data relevant to the reporting frameworks to enable replication in the wider world, should their procedure suggest benefit. The comparison of domain coverage across the two frameworks studied leads us to recommend adherence to the Blencowe framework, as adherence to this is associated with good coverage of the TIDieR framework. This is further supported by the original intent of these tools—TIDieR can be applied across a range of settings including medical therapy and complex interventions, whereas the Blencowe framework is designed specifically for surgical procedures. Consequently, adherence to domains of the latter framework might lead to more applicable reporting for the surgical community. The authors also encourage journal editors to consider adherence to reporting frameworks when publishing future procedure-based studies. The study should also act as a caution to clinicians to consider reporting of techniques critically prior to introduction into practice.

## Conclusions

This study shows that the quality of reporting of surgical procedures to treat Crohn’s anal fistula is limited. Given the significant challenges associated with the treatment of this condition, surgeons must address this reporting to improve study quality, and hopefully outcomes.

## Electronic supplementary material

Below is the link to the electronic supplementary material.

Supplementary material 1 (DOCX 14 kb)

Supplementary material 2 (DOCX 23 kb)
