# Supplementary material for: A systematic review of the quality of reporting of interventions in the surgical treatment of Crohn’s anal fistula: an assessment using the TIDiER and Blencowe frameworks
Source: Tech Coloproctol. 2021 Feb 18;25(4):359–69. doi: 10.1007/s10151-020-02359-7 (PMC8016786; doi:10.1007/s10151-020-02359-7)
Supplement: Supplementary file 1 — Supplementary material 1 (DOCX 14 kb) [file 10151_2020_2359_MOESM1_ESM.docx]

**Appendix A. Example of MEDLINE Search Strategy**

Database(s): **Ovid MEDLINE(R) and In-Process & Other Non-Indexed Citations**1946 to June 07, 2019 
Search Strategy:

| **#** | **Searches** | **Results** |
| --- | --- | --- |
| 1 | Crohn Disease/ | 37134 |
| 2 | Crohn's disease.mp. [mp=title, abstract, original title, name of substance word, subject heading word, floating sub-heading word, keyword heading word, organism supplementary concept word, protocol supplementary concept word, rare disease supplementary concept word, unique identifier, synonyms] | 39450 |
| 3 | Crohn Disease.mp. [mp=title, abstract, original title, name of substance word, subject heading word, floating sub-heading word, keyword heading word, organism supplementary concept word, protocol supplementary concept word, rare disease supplementary concept word, unique identifier, synonyms] | 38388 |
| 4 | 1 or 2 or 3 | 51573 |
| 5 | colorectal surgery/ or general surgery/ | 40919 |
| 6 | general surgery.mp. [mp=title, abstract, original title, name of substance word, subject heading word, floating sub-heading word, keyword heading word, organism supplementary concept word, protocol supplementary concept word, rare disease supplementary concept word, unique identifier, synonyms] | 45667 |
| 7 | surgical procedures.mp. [mp=title, abstract, original title, name of substance word, subject heading word, floating sub-heading word, keyword heading word, organism supplementary concept word, protocol supplementary concept word, rare disease supplementary concept word, unique identifier, synonyms] | 362204 |
| 8 | OTSC.mp. [mp=title, abstract, original title, name of substance word, subject heading word, floating sub-heading word, keyword heading word, organism supplementary concept word, protocol supplementary concept word, rare disease supplementary concept word, unique identifier, synonyms] | 228 |
| 9 | over the scope.mp. [mp=title, abstract, original title, name of substance word, subject heading word, floating sub-heading word, keyword heading word, organism supplementary concept word, protocol supplementary concept word, rare disease supplementary concept word, unique identifier, synonyms] | 526 |
| 10 | surgical instruments.mp. [mp=title, abstract, original title, name of substance word, subject heading word, floating sub-heading word, keyword heading word, organism supplementary concept word, protocol supplementary concept word, rare disease supplementary concept word, unique identifier, synonyms] | 19980 |
| 11 | VAAFT.mp. [mp=title, abstract, original title, name of substance word, subject heading word, floating sub-heading word, keyword heading word, organism supplementary concept word, protocol supplementary concept word, rare disease supplementary concept word, unique identifier, synonyms] | 27 |
| 12 | proctectomy.mp. [mp=title, abstract, original title, name of substance word, subject heading word, floating sub-heading word, keyword heading word, organism supplementary concept word, protocol supplementary concept word, rare disease supplementary concept word, unique identifier, synonyms] | 1240 |
| 13 | fistulotomy.mp. [mp=title, abstract, original title, name of substance word, subject heading word, floating sub-heading word, keyword heading word, organism supplementary concept word, protocol supplementary concept word, rare disease supplementary concept word, unique identifier, synonyms] | 461 |
| 14 | fibrin tissue adhesive.mp. [mp=title, abstract, original title, name of substance word, subject heading word, floating sub-heading word, keyword heading word, organism supplementary concept word, protocol supplementary concept word, rare disease supplementary concept word, unique identifier, synonyms] | 4664 |
| 15 | fibrin glue.mp. [mp=title, abstract, original title, name of substance word, subject heading word, floating sub-heading word, keyword heading word, organism supplementary concept word, protocol supplementary concept word, rare disease supplementary concept word, unique identifier, synonyms] | 3988 |
| 16 | advancement flaps.mp. [mp=title, abstract, original title, name of substance word, subject heading word, floating sub-heading word, keyword heading word, organism supplementary concept word, protocol supplementary concept word, rare disease supplementary concept word, unique identifier, synonyms] | 539 |
| 17 | ligation.mp. [mp=title, abstract, original title, name of substance word, subject heading word, floating sub-heading word, keyword heading word, organism supplementary concept word, protocol supplementary concept word, rare disease supplementary concept word, unique identifier, synonyms] | 78450 |
| 18 | plug.mp. [mp=title, abstract, original title, name of substance word, subject heading word, floating sub-heading word, keyword heading word, organism supplementary concept word, protocol supplementary concept word, rare disease supplementary concept word, unique identifier, synonyms] | 11783 |
| 19 | video assisted.mp. [mp=title, abstract, original title, name of substance word, subject heading word, floating sub-heading word, keyword heading word, organism supplementary concept word, protocol supplementary concept word, rare disease supplementary concept word, unique identifier, synonyms] | 13323 |
| 20 | 5 or 6 or 7 or 8 or 9 or 10 or 11 or 12 or 13 or 14 or 15 or 16 or 17 or 18 or 19 | 524956 |
| 21 | Rectal Fistula/ | 4261 |
| 22 | rectal fistula.mp. [mp=title, abstract, original title, name of substance word, subject heading word, floating sub-heading word, keyword heading word, organism supplementary concept word, protocol supplementary concept word, rare disease supplementary concept word, unique identifier, synonyms] | 4377 |
| 23 | anal fistula.mp. [mp=title, abstract, original title, name of substance word, subject heading word, floating sub-heading word, keyword heading word, organism supplementary concept word, protocol supplementary concept word, rare disease supplementary concept word, unique identifier, synonyms] | 994 |
| 24 | perianal fistula.mp. [mp=title, abstract, original title, name of substance word, subject heading word, floating sub-heading word, keyword heading word, organism supplementary concept word, protocol supplementary concept word, rare disease supplementary concept word, unique identifier, synonyms] | 448 |
| 25 | 21 or 22 or 23 or 24 | 4829 |
| 26 | 4 and 20 and 25 | 240 |
| 27 | limit 26 to (english language and yr="1999 -Current" and english) | 178 |
