# Supplementary material for: A systematic review of the quality of reporting of interventions in the surgical treatment of Crohn’s anal fistula: an assessment using the TIDiER and Blencowe frameworks
Source: Tech Coloproctol. 2021 Feb 18;25(4):359–69. doi: 10.1007/s10151-020-02359-7 (PMC8016786; doi:10.1007/s10151-020-02359-7)
Supplement: Supplementary file 2 — Supplementary material 2 (DOCX 23 kb) [file 10151_2020_2359_MOESM2_ESM.docx]

Appendix B: Risk of bias tables

1a: Non randomised studies

| **AUTHOR** | **YEAR** | **Bias due to confounding** | **Bias in Selection** | **Bias in Classification** | **Bias due to Deviations from Intervention** | **Bias due to missing data** | **Bias in Measurements of Outcomes** | **Bias in Selection of Results** | **OVERALL** |
| --- | --- | --- | --- | --- | --- | --- | --- | --- | --- |
| FY Cheung et al | 2018 | M | M | L | NI | L | M | M | M |
| J.P. Kaminski et al | 2017 | M | M | M | NI | L | M | M | M |
| A Wilhelm el at | 2017 | M | S | M | NI | L | M | M | M |
| Allen B. Dietz et al | 2017 | L | L | L | L | L | M | L | L |
| I. Papaconstantinou et al | 2017 | S | M | L | NI | L | M | M | S |
| R.L Prosst et al | 2016 | S | M | M | L | NI | S | S | S |
| Girolamo Mattioli | 2015 | M | M | M | NI | L | M | M | M |
| R. Mennigen et al | 2015 | M | S | M | NI | NI | M | S | M |
| D. S. Gingold et al | 2014 | M | M | M | NI | NI | S | M | M |
| W.Y Lee et al | 2013 | L | M | L | L | NI | M | M | M |
| O. Schwander | 2013 | L | M | L | NI | NI | M | M | M |
| A. Jarrar et al | 2011 | S | M | S | NI | M | S | C | S |
| G. Owen et al | 2010 | M | S | M | NI | NI | C | M | S |
| W. Chung et al | 2010 | C | M | M | NI | NI | M | M | M |
| V. de Parades et al | 2010 | M | M | L | NI | NI | M | M | M |
| F. de la Portilla et al | 2013 | L | L | L | L | L | L | L | L |
| Bashar Safar et al | 2009 | M | M | M | L | M | M | M | M |
| P.J. van Koperen et al | 2009 | M | M | S | NI | L | M | S | S |
| O. Schwander et al | 2009 | M | M | L | NI | L | S | M | M |
| M. Davies et al | 2008 | S | S | M | NI | NI | M | M | S |
| O. Schwander et al | 2008 | M | M | L | NI | L | M | M | M |
| L. O'Connor et al | 2006 | M | M | M | NI | L | M | M | M |
| S.J. van der Hagen et al | 2006 | S | M | S | NI | S | S | S | S |
| V. Vitton et al | 2005 | M | M | S | NI | L | M | M | S |
| S.M Sentovich | 2003 | S | M | S | L | L | M | M | S |
| S.M Sentovich | 2001 | S | M | S | NI | NI | S | S | S |
| R.L Nelson et al | 2000 | C | S | S | M | M | S | S | S |
| J.J. Park et al | 2000 | S | S | L | NI | L | C | S | S |
| N. Hyman | 1999 | S | M | S | L | NI | S | S | S |
| M. Gautier et al | 2015 | S | M | M | NI | L | M | M | M |

**L = low risk of bias, M = medium risk of bias, H = high risk of bias, C = critical risk of bias, NI = not indicated**

1b: Randomised studies

| **AUTHOR** | **YEAR** | **Random Sequence Generation** | **Allocation Concealment** | **Blinding of Participants and Personnel** | **Blinding of Outcome Assessment** | **Incomplete Outcome Data** | **Selective Reporting** | **Anything Else** |
| --- | --- | --- | --- | --- | --- | --- | --- | --- |
| A. Senejoux et al | 2015 | L | H | H | L | L | L | U |
| J.C Grimaud et al | 2010 | L | H | H | H | L | L | U |
| Panes | 2016 | L | H | L | L | L | L | U |

**L= low risk of bias; S,=some risk of bias; H= high risk of bias, U = unknown risk.**
